# New clues to understand how CENP-A maintains centromere identity

**DOI:** 10.1186/1747-1028-6-11

**Published:** 2011-05-09

**Authors:** Patricia Sánchez, Ana Losada

**Affiliations:** 1 Chromosome Dynamics Group, Molecular Oncology Programme, Spanish National Cancer Research Centre (CNIO), Melchor Fernández Almagro 3, Madrid, E-28029, Spain

## Abstract

The centromere is a specialized chromosomal region that directs the formation of the kinetochore, a huge protein assembly that acts as the attachment site for spindle microtubules and carries out chromosome movement during cell division. Centromere loss or the presence of extra centromeres adversely affect chromosome segregation and may result in aneuploidy, a condition found in many human tumors and a major cause of miscarriages and birth defects. Consequently, understanding the basis of centromere determination and propagation is of great relevance to both fundamental and clinical research. In recent years, it has become clear that centromeres are defined by the presence of a histone H3 variant known as Centromere Protein A, CENP-A, or CenH3. Much effort has been devoted to understanding the mechanisms that drive the assembly of CENP-A containing nucleosomes exclusively onto centromeric DNA, as well as the peculiar structure of these nucleosomes. We have recently developed an immunofluorescence-based assay that measures CENP-A incorporation in the centromeres of chromosomes assembled in *Xenopus *egg extracts. The spatial and temporal specificity of CENP-A deposition observed in human cells can be recapitulated in this in vitro system, making it suitable to dissect the precise role of the different factors that contribute to this pathway. Here, we discuss our results together with other recent advances in our understanding of the mechanisms that mediate centromere inheritance.

## Introduction

Active centromeres are defined by the presence of nucleosomes containing a unique histone H3 variant known as CENP-A [1,2]. Stretched centromeric chromatin from Drosophila, human and chicken DT40 cells shows the presence of interspersed blocks of CENP-A and canonical H3 nucleosomes [3,4]. This chromatin fiber must then be folded to adopt the appropriate compact conformation over which the kinetochore is assembled [5,6]. In proliferating cells, the amount of CENP-A present at centromeres has to be replenished at the time or after centromeric DNA is duplicated in order to propagate centromere identity. The mechanisms responsible for *de novo *CENP-A deposition specifically at centromeres and their regulation in the cell cycle have been an active field of research over the last years. Despite the lack of conservation of centromeric DNA sequences and of the timing of CENP-A nucleosome assembly among different organisms, there is a remarkable conservation of the major players involved in centromere propagation. Thus, research in different experimental systems greatly contributes to put the big picture in focus.

## Discussion

### Mechanisms of CENP-A incorporation mediated by HJURP

**Figure 1:**
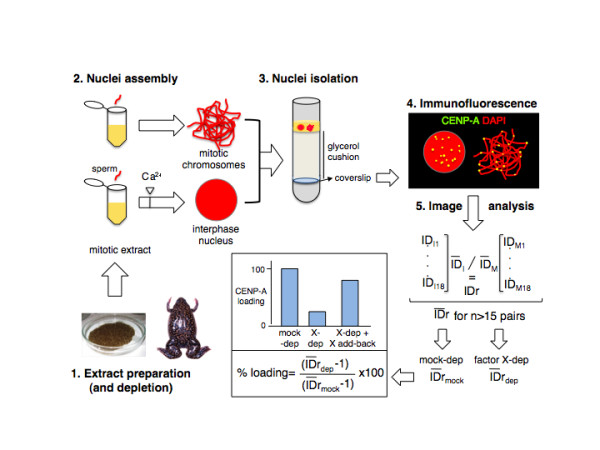
**An assay to measure CENP-A incorporation in Xenopus egg extracts**. Step 1: Extract preparation and depletion. Extracts are prepared from laid eggs arrested in metaphase II (mitotic extracts) and subjected to immunodepletion with specific antibodies against factor X. Step 2: Nuclei assembly. Sperm chromatin is added to mitotic extracts in two different tubes. In one tube (top), incubation proceeds for 80-120 min to get mitotic chromosomes. In another tube (bottom), calcium is added to drive entry into interphase 40 min after sperm addition. Incubation proceeds for 80 min to get interphase nuclei that have undergone replication. Step 3: Nuclei isolation. Equal volumes of the reaction mixtures in both tubes are combined, fixed and centrifuged over a coverslip placed at the bottom of a glycerol cushion. Step 4: Immunofluorescence. Coverslips are processed for immunofluorescence with an antibody against CENP-A and DNA is stained with DAPI. Step 5: Image acquisition and analysis. Images of a mass of mitotic chromosomes next to an interphase nucleus are acquired and CENP-A signals are quantitated using ImageJ software (http://rsb.info.nih.gov/ij/). The average Integrated Density (ID = average pixel intensity × area) is first calculated from the IDs of individual centromeres within each interphase nucleus (ID_I_) and neighboring mass of mitotic chromosomes (ID_M_) and then a ratio between the IDs of each imaged pair is obtained (IDr). Finally, the average ID ratio (IDr) of at least 15 pairs is calculated. The relative CENP-A loading efficiency of a depleted extract with respect to a mock depleted extract (considered 100%) is calculated and represented. If depletion of a factor X prevents loading of CENP-A and the defect is rescued by adding back the factor to the depleted extract, we conclude that factor X is involved in CENP-A incorporation.

One central question of centromere biology is how is CENP-A deposited at centromeric chromatin. Members of a protein family named Scm3/HJURP (Holliday Junction Recognizing Protein), conserved from yeast to humans, have been proposed to act as CENP-A specific chaperones in yeast and human cells [7-15]. These proteins interact physically with CENP-A, can be found at centromeres and, most importantly, are required for CENP-A loading and maintenance. Many additional factors play a role in CENP-A incorporation, but how it actually happens remains unclear. To address this issue, we developed an assay to measure CENP-A incorporation using the Xenopus egg cell-free system [16]. In this immunofluorescence-based assay, nuclei assembled from sperm chromatin and taken at two different time points (e.g. mitosis and subsequent interphase) are combined and processed for immunofluorescence with a CENP-A specific antibody, imaged together, and the centromeric CENP-A signals measured to assess the average difference in intensity between the centromeres within each pair (see Figure 1). We first showed that, in the egg extracts, CENP-A incorporation occurs upon exit from mitosis but independently of DNA replication, same as in Drosophila embryos and human cells [17-20]. Specific immunodepletion of proteins involved in the deposition of other histone H3 variants (CAF-1 and HIRA [21]), did not affect significantly the incorporation of CENP-A. In contrast, we found that CENP-A deposition depends on Xenopus HJURP (xHJURP) and that human HJURP can replace xHJURP. In fact, xHJURP is stored in the oocyte cytoplasm in association with CENP-A, which supports the idea that they form a pre-assembly complex. Analysis of the crystal structure of the CENP-A binding domain of Scm3 in complex with CENP-A and H4 reveals that the interaction of Scm3/HJURP with the CENP-A/H4 dimer is not compatible with binding to DNA further supporting that this is indeed a pre-assembly complex [22].

How HJURP works is still a mystery. It has been recently speculated that its action at centromeres could be coupled to DNA repair [23] or to transcription [24]. In vitro, Scm3 can incorporate CENP-A-containing octamers into plasmid DNA, but not octamers bearing canonical H3 [25]. This probably reflects the fact that the CENP-A targeting domain (CATD), comprised of loop1 and helix 2 of the histone fold domain, not present in H3, is required for the interaction with Scm3/HJURP [14,26]. Importantly, the histone chaperone Nap1 can also do the job (this time for both CENP-A and H3-containing octamers), as it was previously shown for RbAp48 [27]. On the other hand, it is unclear what kind of nucleosomes Scm3/HJURP deposits onto DNA. In one study, nucleosome assembly was not observed upon incubation of Smc3/CENP-A/H4 with plasmid DNA unless H2A/H2B dimers were included in the mixture [25]. However, another group had previously achieved nucleosome reconstitution with this complex and linear lambda DNA [28] and evidence for CENP-A/H4 tetramer deposition on a plasmid template has also been reported for human HJURP [15]. Further studies will be required to explain these controversial results as well as the exact composition of CENP-A nucleosomes *in vivo *(tetrasome, hexasomes, octamers?) and whether they wrap DNA in the conventional left-handed manner or the opposite (for a recent review on these matters, see [29]).

### The role of condensin II in CENP-A deposition and maintenance

Consistent with similar results in human cells, we observed that efficient CENP-A loading during interphase requires previous passage through mitosis [19]. We reasoned that maybe folding of centromeric chromatin during mitosis could be essential for subsequent CENP-A deposition. We thus decided to investigate whether condensin, a major mediator of chromosome condensation, had any role in CENP-A assembly, as suggested by a study in human cells [30]. There are two distinct condensin complexes in most organisms, condensin I and condensin II, which share the SMC2/SMC4 heterodimer but have distinct regulatory subunits. In Xenoupus egg extracts, condensin I is five fold more abundant than condensin II and its role in the assembly of mitotic chromosomes is clearly predominant [31]. However, condensin II accumulates at centromeres suggesting a specific function at this region. Indeed, we found that condensin II has two functions regarding CENP-A assembly. One, it stabilizes CENP-A nucleosomes preventing their eviction. Two, it allows efficient CENP-A incorporation. None of these functions are compensated by the presence of condensin I in extracts lacking condensin II.

The first function refers to the observation that a fraction of CENP-A nucleosomes are lost from centromeres in the absence of condensin II. Easier disassembly of CENP-A nucleosomes compared with canonical H3 nucleosomes could facilitate the clearance of those erroneously incorporated outside of centromeres [32,33] and would be counterbalanced by some property of centromeric chromatin, presumably related to the presence of condensin II in this region. Importantly, this stabilization of CENP-A nucleosomes happens both in mitosis and in interphase.

Regarding the second function, we first suspected that the folding imposed by condensin II could contribute to the recognition of this region by HJURP. However, we did not observe changes in the localization of HJURP in the condensin II depleted extracts. Alternatively, targeting or function of some of the proteins acting downstream of HJURP to effectively incorporate CENP-A might require condensin II. Among those proteins could be remodeling complexes like FACT, RSF1 or CHD1 [34,35] or some of the components of the constitutive centromere-associated network (CCAN) [36,37]. Within this group of inner centromere proteins, CENP-C seems particularly important. On one hand, CENP-C has been proposed to connect CENP-A nucleosomes with CCAN components such as CENP-N [38]. On the other, it links CCAN and the Knl1-Mis12-Ndc80 complex (KMN) network of the outer kinetochore [39-41]. Maybe as a consequence, CENP-C is required to stabilize the folded conformation of the mitotic centromere/kinetochore complex [4]. Since the KMN network, in addition to interacting with microtubules, recruits spindle assembly checkpoint proteins, loss of CENP-C results in multiple, adverse consequences for centromere architecture and function [39,42]. Under this condition, decreased incorporation of CENP-A observed CENP-C knock down cells could be a secondary effect [38]. In this regard, one important advantage of our in vitro assay for CENP-A incorporation in Xenopus egg extracts is that one can analyze the effect of eliminating one specific factor after a single round of CENP-A loading without accumulation of errors from previous rounds or alteration of cell cycle progression. Thus, it will be important to investigate the functional relationship between CENP-C and condensin II in this experimental system.

### How is CENP-A ectopic localization prevented?

Regulation of CENP-A transcription also contributes to specify CENP-A incorporation at centromeres. In human cells, mRNA levels of CENP-A are low during G1 and S phase and increase during G2, prior to its incorporation at the end of the next mitosis [17]. When CENP-A is overexpressed in yeast and Drosophila cells, it incorporates throughout the chromatin [33,43]. However, proteasome-mediated degradation leads to elimination of this mislocalized CENP-A ensuring its exclusive presence at centromeres. Last year, two studies identified an E3 ubiquitin ligase, Psh1, that specifically targets CENP-A in budding yeast [44,45]. Deletion of Psh1 increases CENP-A stability and causes its accumulation at euchromatic regions, but only if CENP-A is highly overexpressed. Like Scm3/HJURP, Psh1 recognizes the CATD of CENP-A. Thus, it is likely that binding of CENP-A to Scm3 at centromeres protects CENP-A from the action of Psh1. Identification of a similar proteolytic regulation in higher eukaryotes remains to be described. In Xenopus egg extracts, protein complexes are stored in the occyte cytoplasm so that the first twelve cycles following fertilization can happen with very little transcription. More than half of the soluble pool of CENP-A molecules in Xenopus egg extracts is bound to HJURP, which could be important to protect CENP-A from degradation. However, we also found that exogenous CENP-A added to these extracts is unable to compete with endogenous CENP-A for the binding of HJURP, but despite this, is stable [16]. Thus, the CENP-A degradation machinery is presumably not operative in the embryonic cycles.

In *Drosophila*, Cal1 was identified in a screening for regulators of the centromeric localization of CENP-A/Cid and CENP-C [46]. More recently, Schittenhelm *et al. *observed that the number of Cal1 molecules per centromere is much lower than CENP-A and CENP-C (2.5, 84 and 135 molecules, respectively) [47]. This, together with the observation that an increase in the centromeric signal of CENP-A is observed only if CENP-A is overexpressed along with Cal1 led the authors to propose that Cal1 restricts the deposition of CENP-A and CENP-C. Whether a similar mechanism exists in other organisms is unclear, since Cal1 orthologs have not been found outside Drosophilids.

### Outlook

Despite recent advances in our understanding of how centromere identity is propagated, in particular the identification of Scm3/HJURP, we are only beginning to elucidate the molecular mechanisms involved and their careful regulation in the cell cycle. Fundamental questions need to be addressed. Does HJURP mediate CENP-A nucleosome assembly by itself or does it require the concerted action of additional chromatin remodelers? What restricts HJURP localization spatially (to centromeres) and temporally (early G1 in the case of human cells)? What is the role of epigenetic marks present on centromeric chromatin? The status of histone acetylation regulated by the Mis18 complex [48] and the presence of H3dimethylK4 [24] have both been proposed to affect CENP-A deposition, presumably by directing HJURP to centromeres, but the exact means of this regulation remains obscure. And what about centromeric RNA transcripts? HJURP and CENP-C accumulate at the nucleolus in interphase, an organelle in which nucleoprotein complexes are assembled [13,49,50]. In fact, CENP-C associates with RNA transcripts derived from centromeric repeats, and these transcripts are required for its centromeric targeting [50,51]. Could HJURP have also an RNA component? Solving these and other exciting questions mentioned throughout this commentary will keep researchers busy in the years ahead.

## Conclusions

Proper assembly of kinetochores at centromeres is essential for accurate chromosome segregation and thereby to ensure genome stability. Centromeres are determined epigenetically by the presence of the histone H3 variant, CENP-A. Several factors implicated in the deposition of this specialized histone have been identified, but the exact molecular mechanism of centromere inheritance remains unclear. We have recently developed an assay that measures CENP-A incorporation at centromeres of chromosomes assembled in *Xenopus *egg extracts and found that this in vitro system recapitulates the temporal restrictions observed in human cells, and that the major players involved, including HJURP, are highly conserved. We are therefore confident that this assay will be a useful tool to further dissect CENP-A deposition in molecular detail.

## Competing interests

The authors declare that they have no competing interests.

## Authors' contributions

PS and AL wrote the manuscript together. Both authors read and approved the final manuscript.
